# Protocol for preparing small, fragile samples from *Schistosoma mansoni* and *Drosophila melanogaster* brains for MALDI mass spectrometry imaging

**DOI:** 10.1016/j.xpro.2026.104789

**Published:** 2026-08-24

**Authors:** Annika Sabine Mokosch, Max A. Müller, Laura Garmendia Sanchez, Stefanie Gerbig, Bernhard Spengler

**Affiliations:** 1 Institute of Inorganic and Analytical Chemistry, Justus Liebig University Giessen, 35392 Giessen, Germany; 2 TransMIT GmbH, 35392 Giessen, Germany; 3 Centre for Neural Circuits and Behaviour, University of Oxford, Oxford, UK

**Keywords:** Chemistry, Organoids, Protocols in Metabolomics and Lipidomics

## Abstract

Mass spectrometry imaging is a routine tool for investigating biological samples with high spatial and mass resolution. However, sample preparation, especially when dealing with very small samples, can be challenging. Here, we describe a sample preparation protocol for small and fragile samples based on examples of *Schistosoma mansoni* parasites and dissected *Drosophila melanogaster* brains. We describe steps for cryosectioning, matrix application, measurement, and data analysis to enable the acquisition of high-quality spatial lipidomics and metabolomics data from small samples.

For complete details on the use and execution of this protocol, please refer to Mokosch et al*.* and Rorsman et al., respectively.^1^^,^^2^

## Highlights

• Modification of a cryosectioning method for small and fragile samples
• Utilizing a gelatin plateau to optimize the sectioning process
• High-resolution mass spectrometry imaging of these small samples
• Visualization and analysis of the acquired data

## Abstract (editor highlights)

Publisher’s note: Undertaking any experimental protocol requires adherence to local institutional guidelines for laboratory safety and ethics.

## Graphical abstract

**Figure:**
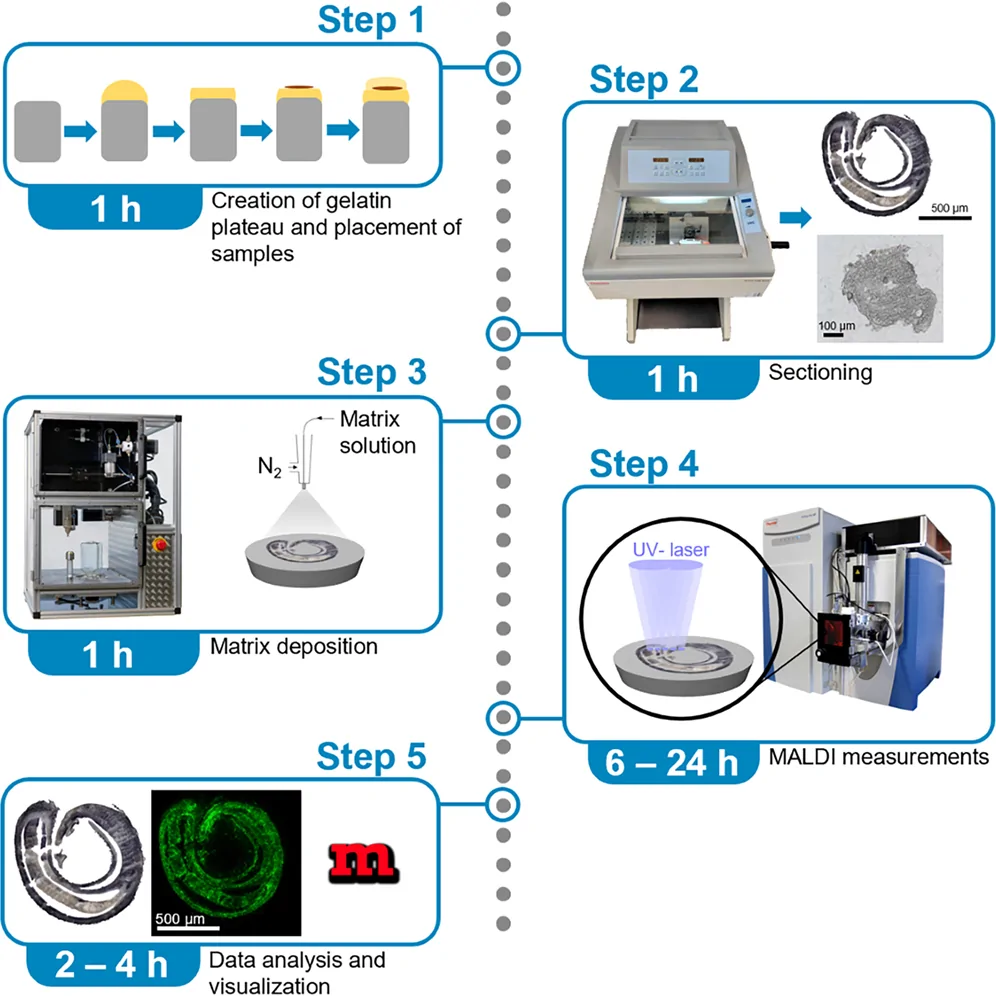

## Before you begin

In recent years, there has been an increased interest in and need for high-resolution measurements and analysis of small samples in the range of 0.1–2 mm length or diameter across the life sciences. This might include, for example, small animals such as certain parasites (e.g., *S. mansoni* worms^3^^,^^4^) or even their distinct organs (e.g., dissected brains of *D. melanogaster* flies^5^). Other typical sources of small samples are organoids, small plants, or their seeds as well as the eggs and larvae of small animals in medical, agricultural, or biological research, respectively.^6^^,^^7^

The investigation of small samples can be important due to the unique threats they pose to human or animal health, such as parasites causing diseases. To develop efficient treatments and identify new drugs to target the spread of these diseases, mass spectrometry imaging can be a useful tool for investigating the metabolism, uptake, and internal spatial distribution of potential drug candidates.^8^ On the other hand, small animals or induced pluripotent stem cell (iPSC)-derived organoids can serve as potent model organisms in multiple research fields, substituting vertebrates like mice for ethical reasons or for addressing personalized medical research. Many small invertebrates, such as *D. melanogaster*, lack the ability to experience pain from experiments and are therefore commonly used models for harsh experiments, eliminating the need for ethical approval.^9^^,^^10^^,^^11^

However, mass spectrometry imaging (MSI), here matrix-assisted laser desorption/ionization (MALDI) MSI,^12^^,^^13^ of small samples poses some major challenges compared to larger samples such as mouse brain. The handling of small samples can be difficult due to their size and fragility, leading to the necessity of embedding the sample in most cases and finding creative ways to transport samples from their source to the sample processing lab without damaging them or changing their morphology. Also, due to their size, there is usually only a small amount of usable sample tissue available, leading to only a small number of possible cryo-sections per sample. To maximize sample yield, it is essential to optimize the sectioning procedure.

The following protocol provides guidance on the process of handling small samples, preparing them for cryo-sectioning, performing MALDI MSI measurements, and analyzing the acquired data. Since high spatial resolution is key for analyzing small samples, we applied an AP-SMALDI^5^ AF ion source with <5 μm laser spot size.^13^^,^^14^ This protocol uses *S. mansoni* parasites as an example of small but tough samples, and *D. melanogaster* brains as an example of especially fragile samples. Both approaches can be readily adapted to other applications.

### Innovation

This protocol describes a modification of the sectioning process for samples used in MALDI MS imaging measurements to adapt the workflow to particularly small samples. It uses embedding in gelatin as the basic method but improves it by integrating the formation of a gelatin plateau on which the samples are placed. The plateau ensures the correct orientation of the samples and results in more sections being obtained in the desired plane. This is especially important for very small or thin samples that do not have enough material or thickness for reorientation during the sectioning process. Another perk of the plateau is the possibility of simultaneous sectioning of two samples for direct comparison.

### Institutional permissions

Breeding of *S. mansoni* parasites requires vertebrate host animals; therefore, institutional approval is necessary for experiments involving these parasites. These experiments have been approved by the Regional Council (Regierungspraesidium) Giessen (V54-19 c 20/15 h 02 GI 18/10 Nr. A 14/2017).

## Key resources table

**Table:** 

REAGENT or RESOURCE	SOURCE	IDENTIFIER
**Chemicals, peptides, and recombinant proteins**		

Gelatin	VWR Chemicals, Radnor, Pennsylvania, USA	CAS No.: 9000-70-8; Cat#24360.233
2,5-Dihydroxybenzoic acid (DHB)	Merck KGaA, Darmstadt, Germany	CAS No.: 490-79-9; Cat#8.41745.0050
Trifluoroacetic acid (TFA)	Merck KGaA, Darmstadt, Germany	CAS No.: 76-05-1; Cat#1.08262.0025
Phosphate-buffered saline (PBS)	Carl Roth GmbH + Co. KG, Karlsruhe, Germany	Cat#:9143.2
Glutaraldehyde Grade I, 25% in H_2_O	Merck KGaA, Darmstadt, Germany	CAS No.: 111-30-8; Cat#G5882
Water	VWR Chemicals, Radnor, Pennsylvania, USA	CAS No.: 7732-18-5; Cat#83645.320
Acetone	Merck KGaA, Darmstadt, Germany	CAS No.: 67-64-1; Cat#1.00022.2500
Liquid nitrogen	Justus Liebig University Giessen, Giessen, Germany	N/A
Dry ice	Justus Liebig University Giessen, Giessen, Germany	N/A

**Experimental models: Organisms/strains**		

Adult *Schistosoma mansoni* couples (liberian strain)^15^ wild type	Lab of Prof. Christoph G. Grevelding, Justus Liebig University Giessen, Germany	N/A
*Drosophila melanogaster* wild type	Lab of Prof. Gero Miesenböck, University of Oxford, United Kingdom	N/A

**Software and algorithms**		

Mirion	TransMIT GmbH, Giessen, Germany	N/A
SMALDIControl v1.6	TransMIT GmbH, Giessen, Germany	N/A
MatrixSprayer v1.39	TransMIT GmbH, Giessen, Germany	N/A
Tune 2.11	Thermo Fisher Scientific, Bremen, Germany	N/A

**Other**		

AP-SMALDI^5^ AF ion source	TransMIT GmbH, Giessen, Germany	https://www.smaldi.de/smaldi-products
Q Exactive HF mass spectrometer	Thermo Fisher Scientific, Bremen, Germany	N/A
Microm™ HM 525 cryotome	Thermo Fisher Scientific, Bremen, Germany	N/A
SMALDIPrep sprayer	TransMIT GmbH, Giessen, Germany	https://www.smaldi.de/smaldi-products
VHX-5000 digital microscope	Keyence, Neu-Isenburg, Germany	N/A
Sample holder (e.g., M6 cylinder screw with hexagon socket and metric thread, flattened tip)	Schraubenhandel Machholz Nchfg. GmbH, Würzburg, Germany	Cat#912-2-6x16/200
Glass slides	Th. Geyer GmbH & Co. KG, Renningen, Germany	Cat#7695003
Sticky tape pads	SPRINTIS Schenk GmbH & Co. KG, Würzburg, Germany	Cat#KPXX000003
IKA MS2 minishaker	IKA Werke GmbH & Co. KG, Staufen im Breisgau, Germany	N/A
IKA MS3 digital	IKA Werke GmbH & Co. KG, Staufen im Breisgau, Germany	Cat#0003319000
PTFE-printed glass slides	Electron Microscopy Sciences, Hatfield, Pennsylvania, USA	Cat#63434-02
Slide mailer boxes	Merck KGaA, Darmstadt, Germany	Cat#HS15983G
MALDI targets	TransMIT GmbH, Giessen, Germany	Cat#TM-PN506
Micropipette 0.1–2.5 μL	Eppendorf SE, Hamburg, Germany	Cat#3123000012
Micropipette 2–20 μL	Eppendorf SE, Hamburg, Germany	Cat#3123000039
Micropipette 20–200 μL	Eppendorf SE, Hamburg, Germany	Cat#3123000055
Micropipette 100–1000 μL	Eppendorf SE, Hamburg, Germany	Cat#3123000063
Micropipette tips 0.1–10 μL	Eppendorf SE, Hamburg, Germany	Cat#0030000811
Micropipette tips 2–200 μL	Eppendorf SE, Hamburg, Germany	Cat#0030000870
Micropipette tips 50–1000 μL	Eppendorf SE, Hamburg, Germany	Cat#0030000919
Microcentrifuge tubes 2 mL	Eppendorf SE, Hamburg, Germany	Cat#0030120094
Centrifuge tube 50 mL	Labcon, Petaluma, California, USA	Cat#3186-345-008
Featherweight forceps	Faude Instruments e.K., Neuhausen ob Eck, Germany	Cat#GF041
Glass cutter	Carl Roth GmbH + Co. KG, Karlsruhe, Germany	Cat#L124.1
Glass pasteur pipette	VWR International, Radnor, Pennsylvania, USA	Cat#612-1702
Dust-free tissue	Kimberly Clark Professional, Roswell, Georgia, USA	Cat#7557

## Materials and equipment

**Table:** DHB matrix solution

Reagent	Final concentration	Amount
2,5-Dihydroxybenzoic acid	30 mg/mL	30 mg
Trifluoroacetic acid	0.1%	1 μL
Water	N/A	500 μL
Acetone	N/A	500 μL
**Total**	**N/A**	**1001 μL**

Store at room temperature and use within a week after preparation.

**CRITICAL: Trifluoroacetic acid:** TFA causes severe skin burns and eye damage upon contact with skin or eyes, respectively. It is also harmful when inhaled. To prevent injuries, avoid breathing mist or vapor and always wear protective clothing, protective gloves, eye and face protection. Only work in well-ventilated areas. If the reagent comes into contact with your skin, immediately take off contaminated clothing and rinse the skin with water. Rinse your eyes cautiously with water for several min after contact, remove contact lenses, if present, and continue rinsing. Move the affected person to fresh air after inhalation of the chemical, keep them comfortable while breathing, and immediately call a poison center or doctor. TFA is also harmful to aquatic life with long-lasting effects; avoid release to the environment.

**2,5-Dihydroxybenzoic acid:** DHB is harmful when swallowed. Wash your skin thoroughly after handling and do not eat, drink, or smoke while working with this product. If you swallow this chemical and feel unwell, call a poison center or doctor.

**Acetone:** Acetone is highly flammable in liquid and vapor form. Keep it away from heat, hot surfaces, open flames, and other ignition sources, and keep the container tightly closed. Ground and bond the container and use explosion-proof electrical, ventilating, and lighting equipment as well as non-sparking tools. Acetone also causes serious eye irritation and can cause drowsiness or dizziness. Repeated exposure can cause skin dryness or cracking. Rinse your eyes for several min after contact, remove contact lenses, if present, and continue rinsing.

***Alternatives:*** It is possible to use alternative matrices instead of DHB for the measurement of samples prepared using this protocol. The choice of matrix depends on the polarity of the ion mode and the investigated molecules. Examples of alternative matrices are α-cyano-4-hydroxycinnamic acid (CHCA) for positive ion mode and 1,5-diaminonaphthalene (DAN) and 9-aminoacridine (9AA) for negative ion mode.

**Table:** Glutaraldehyde solution

Reagent	Final concentration	Amount
PBS	N/A	736 μL
Glutaraldehyde (25%)	6.6%	264 μL
**Total**	**N/A**	**1000 μL**

Prepare fresh every day when needed.

**CRITICAL: Glutaraldehyde:** Glutaraldehyde is harmful when swallowed or inhaled; it can also cause severe skin burns and eye damage. It can also cause an allergic reaction; inhalation can cause allergy, asthma symptoms or breathing problems as well as respiratory irritation. Always use appropriate protective gear, especially gloves and safety glasses. After swallowing glutaraldehyde, contact a poison center or doctor if you feel unwell. If your skin comes into contact with glutaraldehyde, take off all contaminated clothing and rinse the skin with water. Rinse your eyes with water for several min after contact and remove contact lenses. If you inhaled glutaraldehyde, immediately contact a poison center or a doctor and move to a well-ventilated area. Since glutaraldehyde is also toxic to aquatic life and has long lasting effects on aquatic life, avoid release into the environment.

**Table:** Gelatin solution 8 wt%

Reagent	Final concentration	Amount
Water	N/A	42 mL
Gelatin	8 wt%	3.7 g
**Total**	**N/A**	**42 mL**

Store at −20 °C for up to a year.

**Table:** Gelatin solution 5 wt%

Reagent	Final concentration	Amount
Water	N/A	45 mL
Gelatin	5 w%	2.5 g
**Total**	**N/A**	**45 mL**

Store at −20 °C for up to a year.

General safety precautions:

**Liquid nitrogen:** Liquid nitrogen can cause severe cold burns. Handle it with great caution. Always wear protective clothing, especially appropriate gloves. Evaporation of liquid nitrogen can lower the oxygen concentration in the air; this can lead to asphyxiation, especially in confined spaces. Only work in well-ventilated areas. Oxygen sensors can be used as an additional safety precaution in case of a gas spill. Ingestion of liquid nitrogen can cause severe internal damage by freezing the tissue and due to the volume expansion through the warming of the nitrogen.

**Dry ice:** Wear protective gear, especially gloves, when handling dry ice to prevent frostbite. Sublimation of dry ice produces high quantities of carbon dioxide gas, leading to an increased risk of asphyxiation and hypercapnia. Only use it in well-ventilated areas. Since carbon dioxide is heavier than oxygen, it accumulates on the ground and can increase the risk of asphyxiation in those areas.

**Cryotome:** Working with the cryotome poses different risks and thus requires some precautions. The extremely sharp blade is one possible source of injuries; always wear cut-resistant gloves to prevent cuts, especially during cleaning of the cryotome and blade. Another risk is the working temperature of the cryotome; sectioning is done at temperatures between −20 and −25 °C, which can cause harm through frostbite. Wear appropriate protective gloves to prevent frostbite.

**MALDI mass spectrometer:** The mass spectrometer and the coupled MALDI source pose some risks when working with them. The ion source uses two different Class 4 lasers; wear suitable laser safety glasses when working with an open system and restrict access to the room while lasers are running. If you are using a closed system with a safety switch, such as the AP-SMALDI^5^ AF ion source used in this protocol, the built-in safety measures reduce the laser class to Class 1, making the instrument safe to operate without laser safety glasses. High voltages are applied to ion source and mass spectrometer; avoid touching bare wires while using the instrument and ask for technical repair.

The biological samples investigated in this study do not pose a risk of infection or injuries in the form in which they were handled (adult *D. melanogaster* and their dissected brains, as well as adult *S. mansoni* parasites). However, this depends strongly on the type of sample being investigated, choose the protective measures according to the individual sample.

## Step-by-step method details

### Sample-holder preparation

**Timing: 1 h**

This section describes the preparation of sample holders suitable for preparing small samples.

1. Select a screw with a flat top (e.g., an M6 cylinder screw with a hexagon socket and metric thread) with a diameter of approx. 5–7 mm. (Figure 1A)

***Note:*** Ensure that the head of the screw is the correct size and shape to be clamped into the cryotome.

2. Grind down the top of the screw thread with a fine metal rasp or a similar tool until the surface is smooth. The resulting area should have a diameter of at least 5 mm, and it should be an even surface without deep scratches.
3. Mark the head of the screw, either with a pen or by scratching it, so that it can later be fixed in the cryotome in the same orientation.

### Preparation of gelatin solution

**Timing: 3 h**

In this section, the preparation of the gelatin solution is described. Prepare the gelatin solution with great care, as it is a crucial part of the sample preparation protocol.

4. Prepare a solution of gelatin in water with a concentration between 5 and 10 weight percent (wt%) depending on your sample. Consider our examples as a starting point or optimize over different concentrations.
  ***Note:*** The gelatin concentration influences the quality of the sections and is dependent on the sample type, so optimization is necessary. A low concentration (3 wt%) can lead to the formation of a liquid film surrounding the sample and cause metabolites to leak from the tissue into the gelatin. A high concentration (12 wt%) can cause fragmentation of the tissue during sectioning.^16^ Higher concentrations are able to stabilize the sample more, but they also lead to higher volume reduction, and cause fragmentation of the sample through this,^17^ so sample-specific optimization may be necessary. For *S. mansoni* worms, we found the optimal concentration to be 8 wt%, while for *D. melanogaster* brains, the optimal concentration was found to be 5 wt%.
  a. For *S. mansoni*, weigh out 3.7 *g* of gelatin directly into a 50 mL centrifuge tube and add 42 mL of water (HPLC grade).
  b. For *D. melanogaster*, weigh out 2.5 *g* of gelatin directly into a 50 mL centrifuge tube and add 45 mL of water (HPLC grade).
5. Dissolve the gelatin completely, either heat it in a water bath (50–60 °C) or place the centrifuge tube in an ultrasonic bath for approximately 1–2 hours until it is dissolved.
6. Transfer the solution to 2 mL microcentrifuge tubes so that small amounts can be thawed as needed and used for the experiments.
7. Store the solution at −20 °C and thaw it in lukewarm water as needed until the solution has fully liquefied again. It is not required to mix the solution after thawing.

### Collection of *Schistosoma mansoni* samples

**Timing: 1 h**

This section describes the sample collection process for *S. mansoni* samples after extraction from the host animals and optional treatment with drug candidates.

8. First, prepare a solution of glutaraldehyde and phosphate-buffered saline solution (PBS).
  a. Measure 264 μL of glutaraldehyde solution (25%) using a micropipette and transfer to a 2 mL microcentrifuge tube.
  b. Measure 736 μL of PBS using a micropipette and transfer to the microcentrifuge tube.
  c. Thoroughly mix the two solutions using an IKA MS2 minishaker.
  d. Prepare a fresh solution for each sample collection.
9. Label glass slides (e.g., with the sample type, date of sampling, and type of organism).

***Note:*** You can use a pencil or a permanent marker as well as label stickers to label the glass slides on the frosted end of the slides.

10. Prepare a Dewar flask with liquid nitrogen, as well as a holder for microscope slides that allows them to be immersed in the liquid nitrogen.

**Caution:** Liquid nitrogen can cause serious injuries, so follow the appropriate safety precautions. These include wearing safety glasses, a lab coat, and gloves as well as working in well-ventilated rooms.

11. Transfer approximately 50–100 μl of the glutaraldehyde-PBS solution to one of the labeled slides.
12. Remove the living *S. mansoni* worms from their culture solution using featherweight forceps and place them in the glutaraldehyde solution on the glass slide.
  ***Note:*** The worms can either be paired worms or single worms (male or female).
  a. Place approximately 10 worms treated in the same way in the solution on each slide. The number of samples per glass slide can be adapted according to the sample type.
13. Immerse the slide with the worms in liquid nitrogen for a few seconds.
  a. Completely submerge the slide, including the worms.
  b. Remove the slide from the liquid nitrogen once the glutaraldehyde-PBS solution turns white and the boiling of the liquid nitrogen has stopped.
14. Store the samples at −80 °C prior to sectioning.

### Collection of *Drosophila melanogaster* samples

**Timing: 1 h**

In this section, the collection of *D. melanogaster* brains is described.

15. Collect *D. melanogaster* in vials and anesthetize them by placing vials on ice.
16. Dissect their brain on standard phosphate-buffered saline (PBS) medium.^18^
17. First, prepare a small amount of PBS in an individual well of a PTFE-printed glass slide with a pipette to avoid the brain from getting stuck to the glass surface, which would prevent positioning of the brain in the correct orientation.
18. Transfer the brain onto the glass slide using a glass pipette by gently aspirating the brain around the tip of the pipette and dispensing it into the well.

**Caution:** Perform this step very gently to avoid the brain getting stuck inside the glass pipette.

***Optional:*** Alternatively, transfer the brain by using the dissection forceps and gently place the brain on the tips of the forceps. However, this approach is more prone to damage the brain structure.

19. Once the brain is in the slide well, use the dissection forceps to position the brain in the correct orientation.

***Note:*** All brains should be positioned in the same orientation (e.g., ventral to dorsal, anterior to posterior). Any variations in orientation should be annotated.

20. Once positioned appropriately, use a pipette with a small tip to aspirate as much of the PBS liquid as possible from the well.
  **Caution:** Perform this step very gently without directly touching the brain with the pipette tip to prevent it from sticking to the plastic surface.
  a. Check whether the brain remains at the bottom of the well and in the appropriate position.
21. Immediately cover the brains with 3–5 μL of gelatin.
22. Immerse the slide with the brains in liquid nitrogen for a few seconds.
  a. Completely submerge the slide.
  b. When the boiling of the liquid nitrogen stops, remove the slide from the liquid nitrogen.
23. Store the samples at −80 °C or ship them on dry ice.
  Caution: Dry ice can cause injuries, so follow safety precautions, including wearing protective gear and working in well-ventilated areas.
  a. Prevent movement and damage of slides during storage and transportation by placing the glass slides into slide mailer boxes.
  b. Place a small piece of flexible material (e.g., flexible polyurethane foam) between the slides and the box opening to fill the gap, this serves as a cushion to prevent movement and impact on the slides.
  c. Seal the mailer boxes with tape to prevent opening during shipping.

### Cryosectioning: Preparation of the gelatin support

**Timing: 0.5 h**

This section describes how to perform cryosectioning to obtain high-quality sections from small samples such as *S. mansoni* parasites or dissected *D. melanogaster* brains. By using a gelatin plateau and careful handling, precise sections with a thickness of 10–40 μm can be obtained while preserving the anatomical features of the tissue.

24. Place the container with the solidified gelatin in warm water for 5–10 min to liquefy it.
25. Using a micropipette, draw up 20 μL of the gelatin solution and apply it to the surface of the sample holder. (Figure 1B)
  a. A hemispherical gelatin dome will form. (Figure 1C)
  b. Ensure that there are **no air bubbles in the gelatin** dome. (Troubleshooting Problem 1)
  c. Increase the amount of gelatin to 30 μL if a larger plateau is required, for example, to section two samples at the same time.

**Figure 1:**
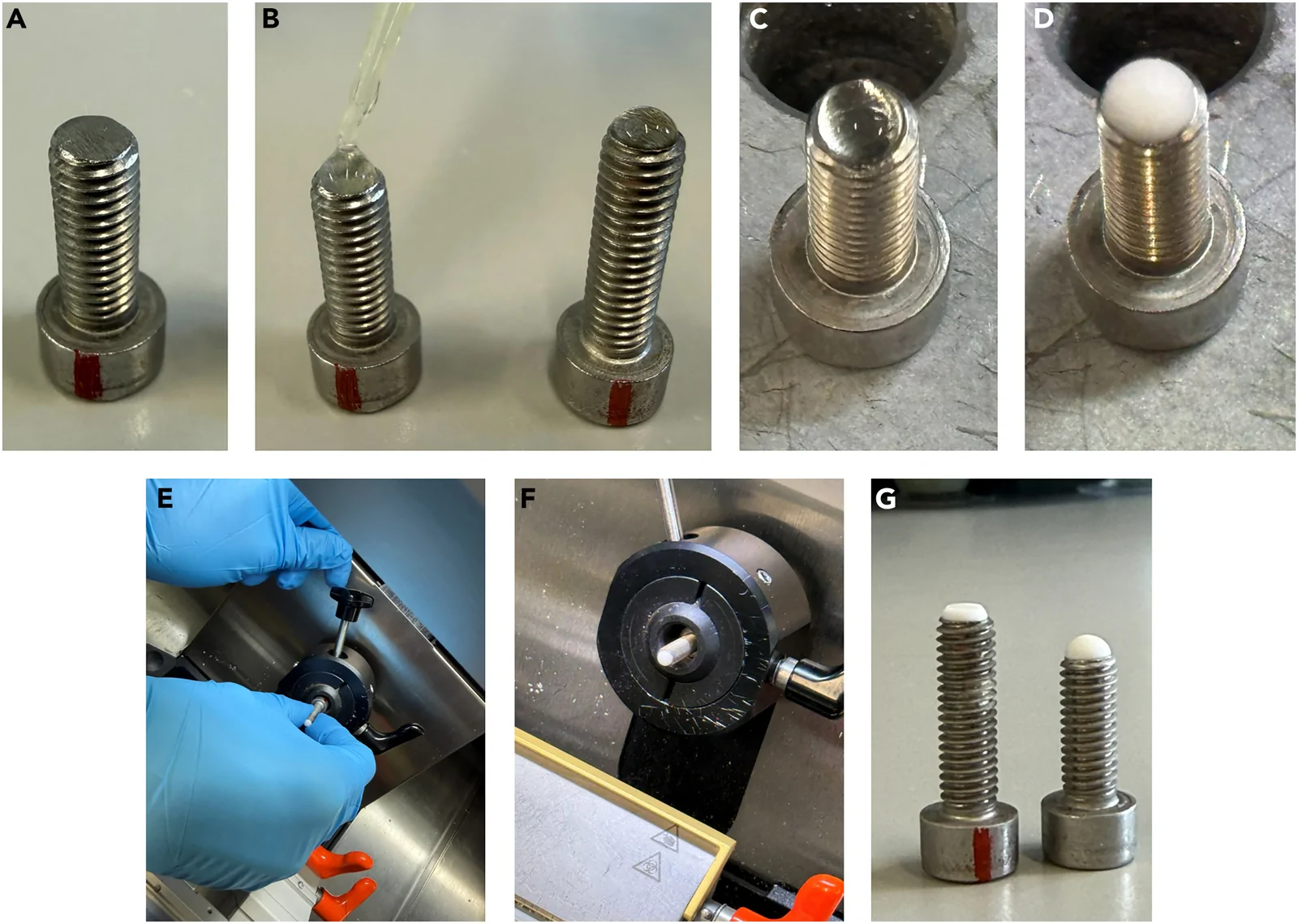
Preparation of the gelatin plateau (A) Sample holder with marking on the head of the screw. (B) Placing 20 μL of gelatin on top of the sample holder. (C) Gelatin drop on sample holder prior to freezing. (D) Frozen gelatin drop on top of the sample holder. (E) Placing the sample holder in the cryotome with the red marking facing upwards. (F) Sample holder in the cryotome ready to section the gelatin. (G) Created plateau on the left sample holder in comparison to the frozen gelatin drop.
26. Place the sample holder in the cryotome for 5–10 min at −25 °C to allow the gelatin to freeze through.
  a. The gelatin is frozen through when it becomes opaque. (Figure 1D) (Troubleshooting Problem 2).
27. Secure the sample holder in the holder with the **marking facing upwards.** (Figures 1E and 1F)
28. Set the section thickness of the cryotome to 50–100 μm and begin making sections until a sufficiently large, flat area of the gelatin has been created.

**Caution:** Very sharp blade! Handle the blade carefully; do not touch it and wear protective gloves, especially during cleaning.

29. Use a soft brush to remove any sections from the cryotome blade and the resulting gelatin plateau.
30. Store the sample holder in the cryotome until it is needed again. (Figure 1G)

### Cryosectioning: Transfer of *Schistosoma mansoni* samples

**Timing: 1 h per sample**

This section describes the workflow for transferring *S. mansoni* parasites to the gelatin plateau.

**Figure 2:**
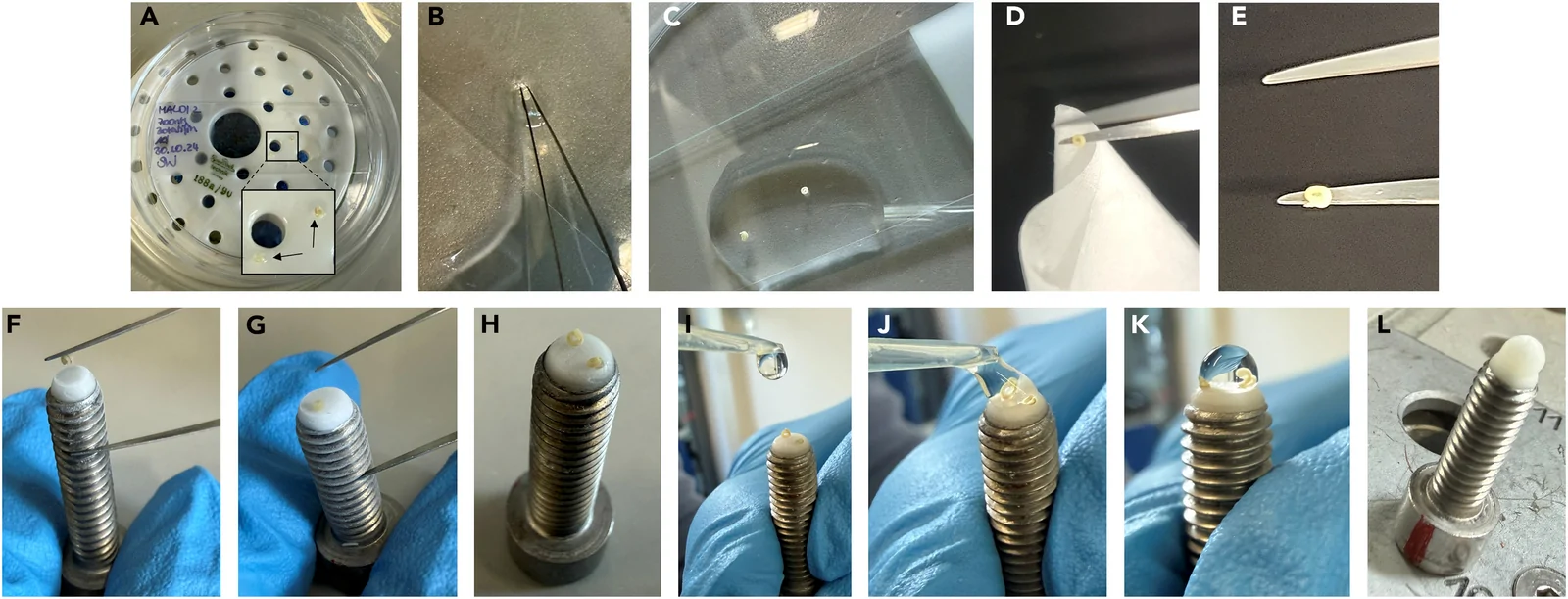
Transfer of *Schistosoma mansoni* parasites to the gelatin plateau (A) *S. mansoni* worms on glass slide in desiccator. (B) Taking one *S. mansoni* worm couple from the glass slide using featherweight forceps. (C) Putting the *S. mansoni* worm couple into water on a glass slide for approximately 20 seconds. (D) The excess water is carefully absorbed with a tissue. (E) The worm pair on the featherweight forceps. (F) Placing the worm pair on the gelatin plateau on the sample holder. (G) The worm pair is placed on the gelatin plateau. It is helpful to secure the forceps by placing the lower arm on the thread of the sample holder screw and then tipping the upper arm with the worm couple slightly on the surface of the gelatin. (H) Two *S. mansoni* couples on the gelatin plateau. (I) After cooling down the worm pair on the plateau in the cryotome, place a drop of gelatin (20 μL) on top. (J) Let the gelatin drop hang from the side of the micropipette and carefully place the drop over the worm sample. (K) The worm sample inside the drop of gelatin. (L) The frozen gelatin including the worm sample, which is not visible due to the frozen gelatin.
32. Meanwhile, place a glass slide in a Petri dish and apply approximately 100 μL of water (HPLC grade) on top.
33. If the prepared gelatin solution in the microcentrifuge tube has solidified, liquefy it again in warm water.
34. After thawing, place a pair of worms in the water on the glass slide using featherweight forceps. (Figures 2B and 2C) (Troubleshooting Problem 3 and Problem 4)

***Note:*** This step is carried out to remove the remaining glutaraldehyde/PBS solution to reduce the formation of bubbles in the gelatin surrounding the sample.

31. Place the samples that have been stored at −80 °C in a desiccator at room temperature (approx. 10 min). (Figure 2A) The collection of these samples is described in the first part of this protocol under “Collection of *Schistosoma mansoni* samples”.

35. Take the sample carrier out of the cryotome and place it next to the microscope slide.
36. Remove the worm from the water with featherweight forceps after about 10 seconds and wipe off excess water with the tip of a dust-free tissue. (Figure 2D)
37. Place the worm on the gelatin plateau using the forceps.
  a. Hold the lower arm of the forceps against the sample holder to stabilize the forceps while gently tapping the upper arm holding the worm onto the gelatin plateau. (Figures 2F and 2G)
  b. Perform this step quickly to prevent the gelatin from becoming too warm.

***Optional:*** You can also take two pairs of the *S. mansoni* worms, and place them next to each other, enabling the simultaneous measurement of a treated and an untreated sample, or a genetically modified worm pair and a control pair, for example. For the placing of two different samples, place the sample holder with the first sample in the cryotome to cool it down again while preparing the second sample following the steps 3137.

38. Place the sample holder containing the worms back into the cryotome at −25 °C and cool for approx. 2 min to allow the worms to firmly attach to the surface.
39. Using a micropipette, draw up 20 μL of the gelatin solution. Hold the drop on the side of the tip and then carefully place the gelatin (8 wt%) over the worm. (Figures 2H, 2I, and 2J)
40. Place the sample holder back into the cryotome, allowing the gelatin to fully freeze (approx. 3–5 min). (Figure 2K)

### Cryosectioning: Transfer of *Drosophila melanogaster* samples

**Timing: 0.5 h per sample**

The transfer of *D. melanogaster* brains to the gelatin plateau is described in this section.

41. Place the samples that have been stored at −80 °C in a desiccator at room temperature (approx. 10 min). The collection of these samples is described in the first part of this protocol under “Collection of *D. melanogaster* samples”.
42. If the prepared gelatin solution in the microcentrifuge tube has solidified, liquefy it again in warm water at this step.
43. Cover the brains in 20 μL of liquid gelatin solution (5 wt%) using a micropipette. Do not release the droplet and do not remove the micropipette. (Figure 3B)

**Figure 3:**
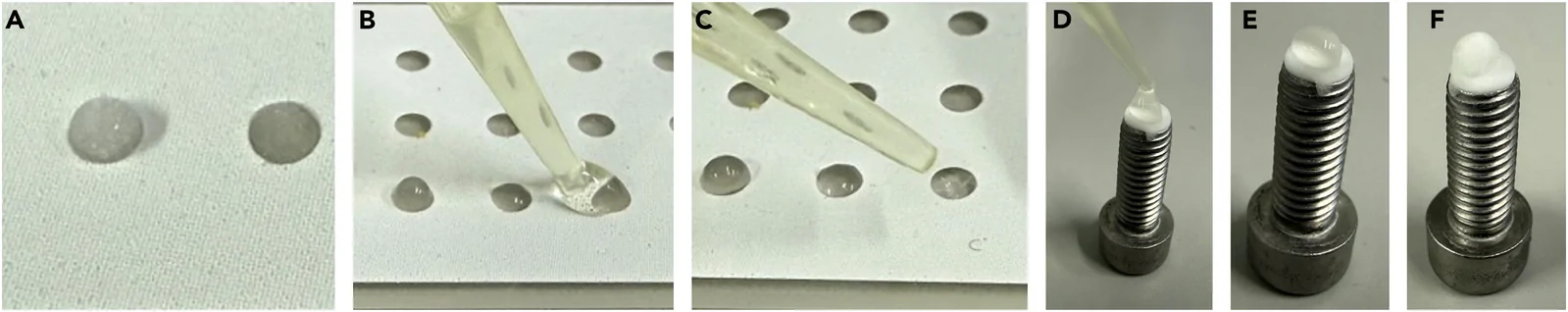
Transfer of *Drosophila melanogaster* brains to the gelatin plateau (A) The sample (*Drosophila melanogaster* brains) after being taken out of the freezer. (B) A drop of gelatin is applied to the selected sample. (C) The gelatin is pipetted back up immediately. (D) The gelatin drop is placed on the gelatin plateau on the sample carrier. (E) The gelatin drop containing the *D. melanogaster* brain is located on the gelatin plateau. (F) All of the gelatin is frozen, including the *D. melanogaster* brain inside.
44. Place the pipette tip near the brain and aspirate the gelatin back into the micropipette. This will carry the brain with it. (Figure 3C) (Troubleshooting Problem 5).
45. Check the glass slide to determine whether the brain has been successfully transferred into the micropipette.
46. Place the sample-containing gelatin solution onto the gelatin plateau inside the cryotome. The sample will sink toward the bottom of the droplet. (Figures 3D and 3E)
47. Allow the gelatin to fully freeze (approx. 3–5 min). (Figure 3F)

### Cryosectioning: Preparation of sections

**Timing: 1–2 h**

This section describes the process of sectioning the samples embedded in gelatin.

48. Clamp the sample holder into the bracket and ensure that the marking is facing upwards. (Figures 4A and 4B)

**Figure 4:**
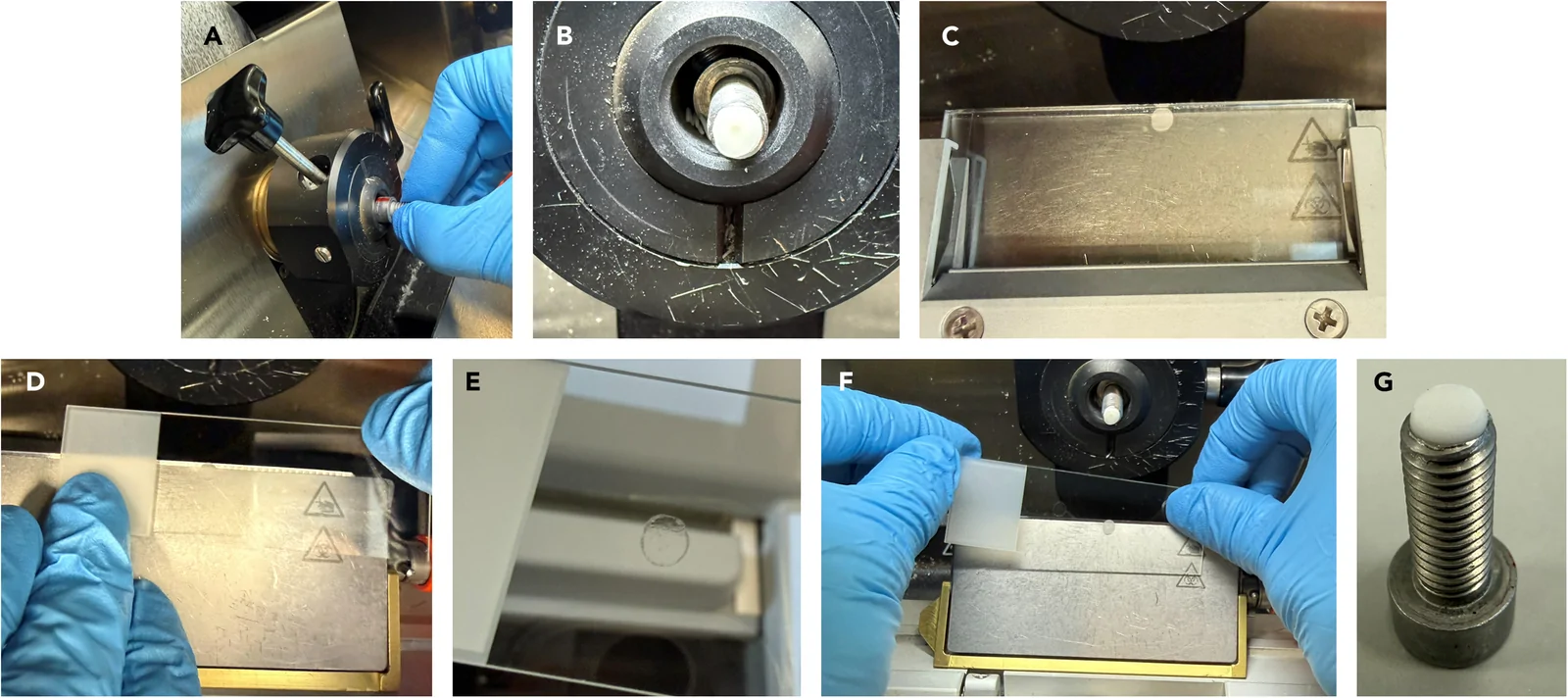
Cyrosectioning (A) Placing the sample holder back in the cryotome with the red marking facing upwards. (B) The sample holder is fastened into the cryotome. (C) A section of the gelatin is located under the anti-roll glass plate of the cryotome. (D) Taking up the section with the glass slide, lowering the glass plate from the right side. (E) The section is placed on the glass slide. (F) Taking up the next section with the same glass slide but lowering the glass plate from the left side to protect the first section. (G) The sample carrier after the sectioning, with some gelatin left.
49. Set the section thickness to the desired value (10–40 μm).
50. Move the cryotome handle clockwise to obtain sections of the desired thickness.
51. At first, you only section gelatin. (Figure 4C).
  a. As you get closer to the worm, the gelatin changes slightly and a kind of imprint of the sample appears in the sections.
  b. As soon as you get closer to the sample, take test sections on a microscope slide to avoid missing the sample. Check by eye or with a microscope whether you are sectioning the sample or pure gelatin.
52. To capture the sections, take a glass slide and press it with the side bearing the label facing down onto the surface where the section is located. (Troubleshooting Problem 6).
  a. Press the left side of the slide onto the surface with your thumb, while slowly guiding the glass slide downwards with your right hand until the section is on the glass slide.
  b. If several sections are to be placed on one slide, press the right side onto the surface with your thumb from the second section onwards.
  c. Then slowly guide the left side down to avoid damaging the sections already on the glass slide. (Figures 4D and 4F).
53. The cutting area and the blade should be cleaned with a soft brush between sections.
54. Once only gelatin slices remain, the sample is completely sectioned. (Figure 4G).
55. The sections can be used immediately after sectioning or stored at −80 °C.

### Matrix deposition

**Timing: 1 h**

This section describes how to apply the matrix to the sample using a SMALDIPrep sprayer (TransMIT GmbH) to ensure a uniform, partially automated application of the matrix. The uniform matrix layer facilitates high-resolution measurements, regarding both spatial and mass resolution, even for samples with low analyte concentrations. The following workflow is based on our sample preparation routine and can be adapted depending on the sample type, the matrix used, and the sprayer system.

56. Weigh out 30–60 mg of DHB directly into a 2 mL microcentrifuge tube. The matrix application can be adapted regarding concentration, amount of matrix, solvent composition, and spray flow.

***Note:*** We used DHB for our measurements, because our main focus in these experiments was the detection of lipids. The use of other matrices is also possible; choose them depending on the target molecules. Examples of matrices and alternative matrix deposition methods are shown by Wu *et al.*^19^

57. Use a spreadsheet to calculate the amount of solvent needed to obtain a solution with a concentration of 30 mg/mL. The exact recipe for 1 mL of this solution is described under materials and equipment.
  a. Water and acetone are used as solvents in a 1:1 (v/v) ratio.
  b. Add Trifluorocacetic acid (TFA) to the solvent mixture to achieve a final concentration of 0.1 volume percent (vol%).
58. Measure the calculated amounts of water, acetone, and TFA using micropipettes and add them to the DHB in the microcentrifuge tube.
59. Thoroughly mix the solution using an IKA MS3 digital shaker.
60. Take the sectioned sample out of the freezer and place it immediately in a desiccator to warm up to room temperature for approx. 30 min.
61. Cut the glass slide using a glass cutter so that the piece with the sample to be measured remains.
62. Attach the sample to a target using a piece of adhesive tape.
63. Place the sample target onto the sample holder in the SMALDIPrep matrix sprayer.
64. Fill the syringe with the freshly prepared matrix solution.
65. Start the SMALDIPrep software and initialize all components as requested.
66. Select “HighRes” as application mode.
67. Select a predefined method or adjust parameters for optimal spraying results. Apply DHB matrix with a flow rate of 5 μL/min for 30 min and nebulize the spray with a nitrogen flow of 5 L/min. (Troubleshooting Problem 7 and Problem 8).
  a. The optimal spraying parameters are highly dependent on the matrix, solvent composition, and sample type and can also be influenced by the temperature or humidity of the environment. Optimize these parameters individually when reproducing the results presented in this paper.
68. Remove the sample and check the matrix under a microscope for the correct crystal size and a uniform coating.

***Note:*** A uniform coating of the matrix is essential for obtaining high-quality MSI data. Gaps in the matrix layer can result in incomplete images. Large chunks of matrix may lead to ion suppression, as matrix-derived signals can dominate over analyte signals. Meanwhile, an insufficiently thick matrix layer can cause an overall decline in signal intensity. A similar effect may be caused by poor co-crystallization due to a negative impact on the ionization efficiency. These challenges highlight the importance of careful and consistent sample preparation and maintaining stable environment conditions during matrix application. It is also very important to follow an appropriate cleaning routine for the sprayer to prevent clogging or matrix accumulation.

69. Measure the sample immediately or store it for up to 2 days under atmospheric conditions, as the matrix protects the analytes from rapid deterioration.

### MALDI measurements

**Timing: 6**–**24 h**

This section describes the measurement method, which uses MALDI MSI to generate spatially resolved mass spectrometric data. An AP-SMALDI^5^ AF ion source (TransMIT GmbH) coupled with a Q Exactive HF Orbitrap mass spectrometer (Thermo Fisher Scientific) enables high mass resolution as well as high spatial resolution.

70. Insert the matrix-coated sample into the MALDI target holder.
71. Insert the sample holder into the AP-SMALDI^5^ AF ion source.

***Optional:*** Calibrate the stage of the ion source by opening the menu under “Device” and selecting “Calibrate Stage”.

**Caution:** The AP-SMALDI^5^ AF ion source uses lasers for the autofocus and the ionization as well as high voltage. Do not bypass the system’s safety features and do not open the protected housing without authorization.

72. Use the autofocus function in the SMALDI control software to focus on the correct plane of the sample.

***Note:*** Check the autofocus manually every week by observing the ablation spots with a microscope to ensure correct settings.

73. Optimize the laser energy applied to the sample via test shots on the sample or the surrounding matrix layer. Find a good compromise between signal intensity (in the mass spectrometer) and ablation spot size (as observed under a microscope).

***Note:*** Increasing laser energy yields higher signal intensities, but increases in-source fragmentation, which negatively affects sensitivity of labile analytes. Further, it increases the ablated area on the sample surface, which can cause issues with lateral resolution on very small samples, leading to oversampling by ablating already measured areas of the sample.

74. Use the SMALDIControl software to select a rectangular area covering the entire sample.
75. Set the step size to 5–10 μm, depending on the desired lateral resolution and the available measurement time. The desired lateral resolution should be lower than or equal to the individual laser ablation spot size of the instrument to avoid oversampling.

***Note:*** The measurement duration depends on the spatial resolution at a given the sample size. Measurements of *S. mansoni* sections using a step size of 5 μm take approximately 14–18 h when measuring one worm pair in step-by-step mode. For the simultaneous measurement of two pairs with a step size of 10 μm, plan at least 24 h of measurement time.

76. Use the standard 2D mode for flat samples. For samples with significant height variations, enable the autofocus during the measurements by selecting 3D mode.
77. Set the method parameters in the mass spectrometer’s Tune software as follows (Table 1):
  a. Adjust the mass range according to the analytes being investigated. It should not exceed *m*/*z* x to 4x to avoid split injection of ions.
  b. Select the lock mass according to the matrix used. It should be located roughly in the middle of the measured mass range.

**Table 1:** Method parameters for MSI measurements

Setting	Value
Scan type	Full MS
Scan range	250 to 1,000 *m*/*z*
Fragmentation	None
Resolution	240,000
Polarity	Positive
Microscans	1
Lock Mass	716.12461 (DHB cluster, [5M + NH_4_-4H_2_O^+^])^20^
Spray Voltage	3.00 kV
Capillary Temp.	250 °C
S-lens RF level	100
78. Enter the file name in the Tune software, including the sample ID, date, step size, number of pixels in x and y directions, mass range, and ion mode.
79. Select “Start” in the Tune software. (Troubleshooting Problem 9)
80. Select “Measure” in the SMALDI control software.
81. After the measurement is complete, you obtain a raw-file by the Tune software and an UDP file by the SMALDIControl software. Use these files for image generation and data analysis.

### Data analysis and visualization

**Timing: 2**–**4 h**

This section describes the analysis of the measured data and the creation of images using the Mirion^21^ software package. The acquired data can be used to generate ion distribution images. When 3D mode is used during measurement, the topographic profile of the sample can be displayed simultaneously with the ion distributions.

82. Launch the Mirion software (mirion 3D V3.4.64.21).
83. Select “File,” then “New Project” to select the UDP file of the measurement. Confirm your selection by clicking “Ok”. (Figures 5A, 5B, and 5C)

**Figure 5:**
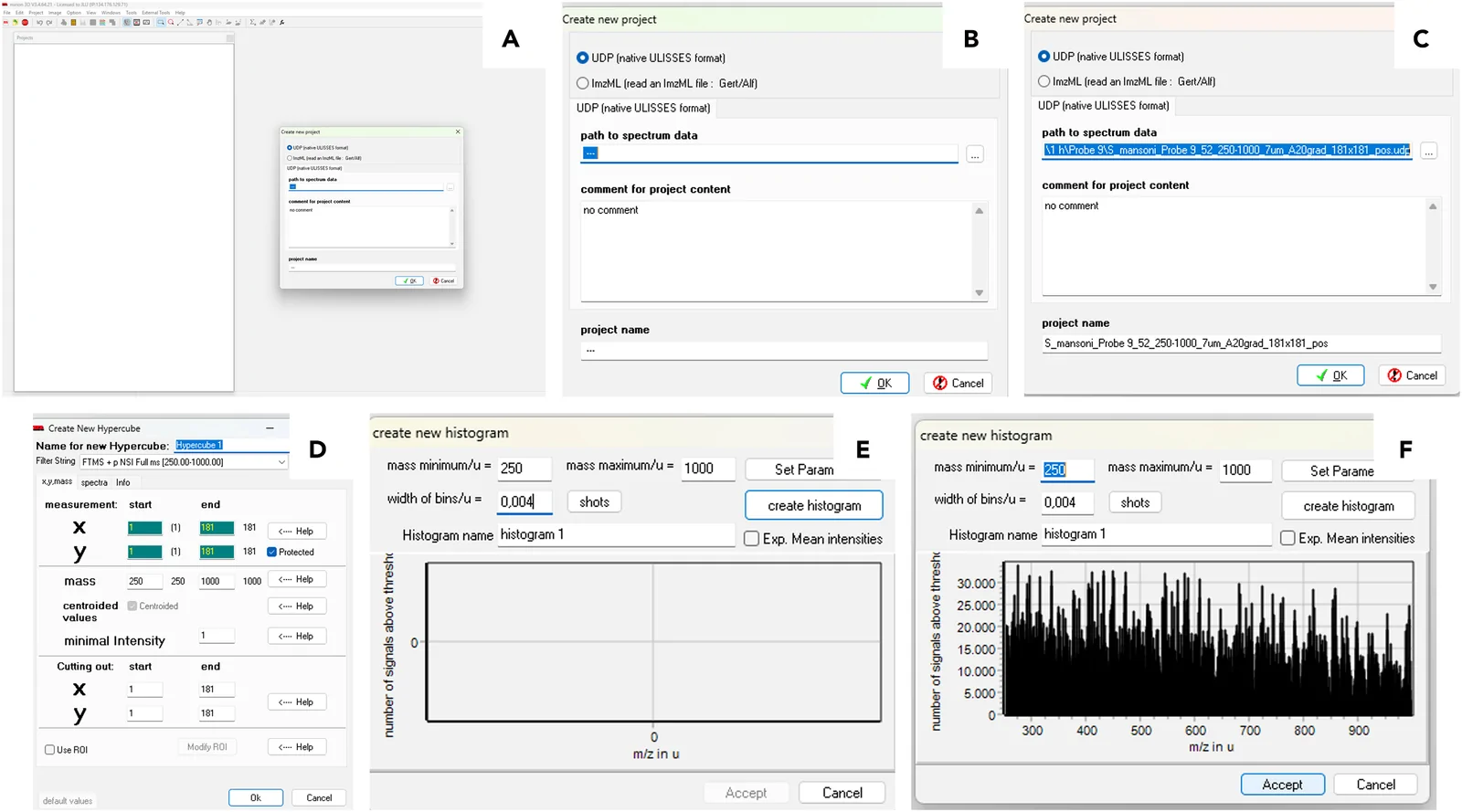
Data processing and generation of the histogram using Mirion (A) The Mirion program has been launched and the window for selecting the measurement file is displayed. (B) The window for selecting the measurement file is now displayed. (C) The window for selecting the measurement file is now displayed with the selected measurement. (D) A window for creating the hypercube is displayed. (E) The window for creating the histogram is shown. (F) The generated histogram is displayed and can now be confirmed.
84. Right-click the project name in the sidebar and select “New Hypercube” from the menu to create a hypercube. (Figure 5D)
  a. The UDP file defines the number of pixels in x and y as well as the mass range.
85. Click “Ok” to create the hypercube. This process can take several min to complete. The hypercube rearranges the data in the computer’s memory to enable fast image creation.

***Optional:*** Select the “Use ROI” checkbox to manually define a region of interest (e.g., the sample or specific parts of the sample). The region of interest can be of any shape and defines the pixels included for further processing.

**Figure 6:**
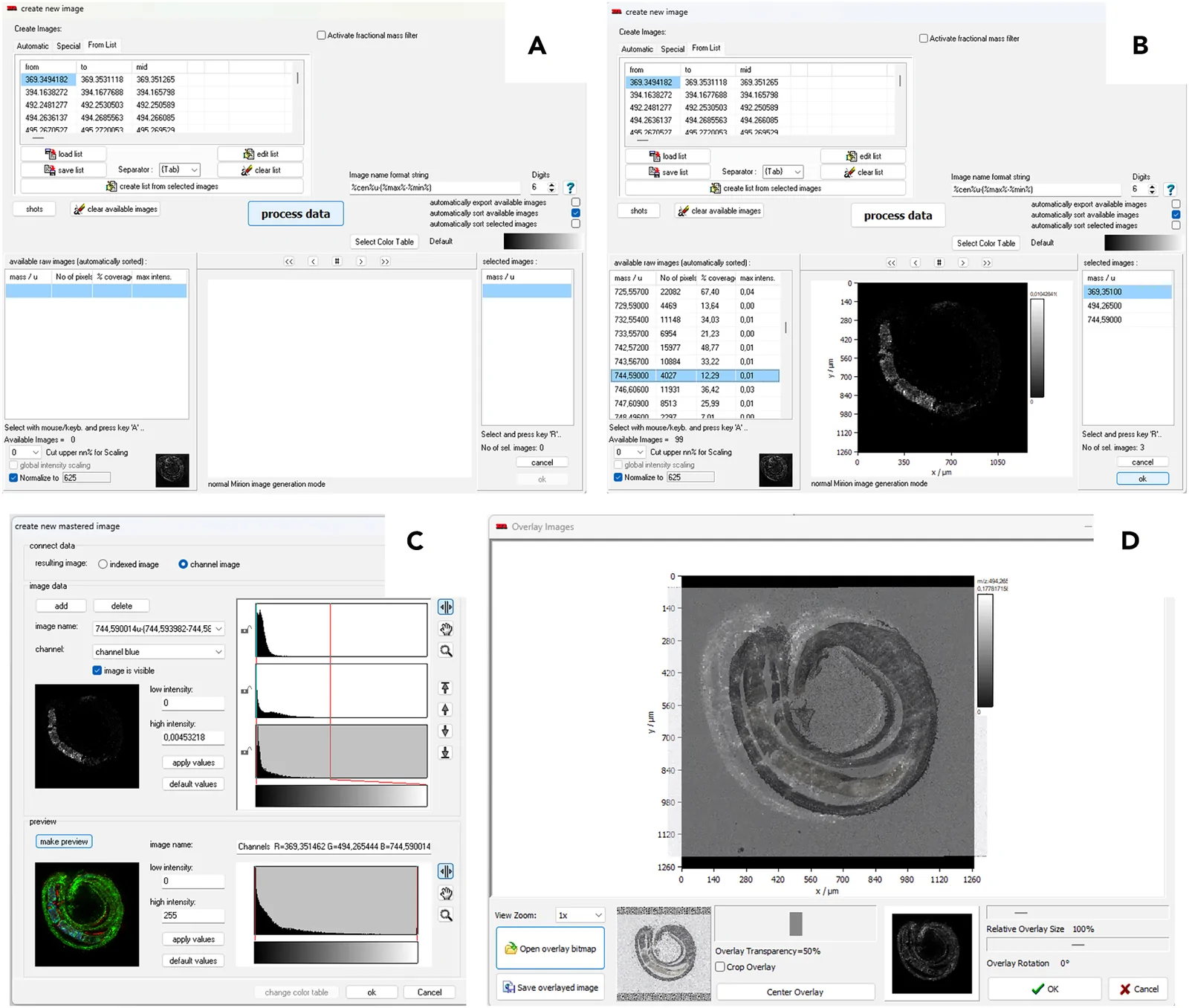
Image generation with Mirion (A) The image creation window is displayed, with a list of interesting masses already selected. (B) Now you can select distribution images of different masses so that they can be generated in the next step. (C) Overlay of three selected m/z ratios, with each distribution assigned its own color (red, green, blue). (D) Overlay of distribution image of an *m*/*z* ratio and the optical image, immediately after selection of the optical image.

86. Select the hypercube in the sidebar, right-click it, and select “New Histogram” to open the dialogue box.
87. Set the value for “width of bins/u” to 0.004. (Figure 5E)
88. Select “Create histogram” to generate the histogram. After the process is complete, click “Accept” to proceed. (Figure 5F)
89. Select the histogram from the sidebar, right-click it, and Select “New Image” to open the image-creation menu.
90. The image-creation menu allows for automatic, targeted or list-based image creation. (Figure 6A)
  a. For a first overview of the sample, select “Automatic” to create images of the most intense signals. Adjust the parameters as needed. In most cases, the default settings are sufficient.
  b. If a list of potential analytes is already available, select “From List” to display only those images. Load a list containing information on the analytes of interest (“mz mid”) and the desired mass window (“mz from” and “mz to”).

***Optional:*** Select the “Normalize to” checkbox to normalize the images to the total ion count.

***Optional:*** Click “Select Color Table” to change the color gradient used to display the intensity values in the images. Standard is greyscale.

91. Click “Process Data” to generate images defined in the previous steps. The generated images will be listed below.
92. Browse the available images and manually select the images of interest by clicking the “>” button above the image or select all generated images by clicking the “>>” button. Only the images selected here will be available for further processing! (Figure 6B)

***Optional:*** Save the secondary data (mass window, accurate mass, image coverage, and more) of the selected images as a list using the tab “from list”, then click “create list from selected images” followed by “save list” for later use.

93. Click “ok” to transfer the selected images into the Mirion main window for further processing.
94. Click the “Export Single Image” icon in the image window of an individual image to export it to your computer. Add information on sample ID to the standard filename of the image. RGB-images can be created from overlaying up to three greyscale images, or the images can be overlaid with the optical image.
95. To create an RGB-image, select one of the desired images from the sidebar, right-click it, and select “New Mastered Image” from the menu.
  a. Add up to two additional greyscale images using the “Add” button.
  b. Adjust the balance between the three colors using the histogram sliders.
  c. Click “Make Preview” to generate a preview, then generate the final image by clicking “ok”. (Figure 6C)
96. To overlay an image with an optical image, select the desired image from the sidebar, then click “Project” in the menu and select “Overlay Images.”
  a. Select the optical image in the window that opens by clicking on “Open overlay bitmap.”
  b. Adjust the size, angle, and transparency of the optical image as required to fit to the ion image, then save to the hard drive of the computer by clicking on “Save overlayed image”. (Figure 6D)

## Expected outcomes

This protocol enables users to section small and fragile samples in their native state without chemical fixation, producing damage-free sections while preserving structural morphology and chemical composition. This is crucial for generating information-rich, high-resolution mass spectrometry images to investigate the distribution of lipids, metabolites, and drug candidates. By applying this protocol, users can expect to gain information on the semi-quantitative distribution of hundreds of analytes simultaneously from a single measurement. By providing different examples, this protocol enables readers to adapt it to different hard-to-handle samples.

**Figure 7:**
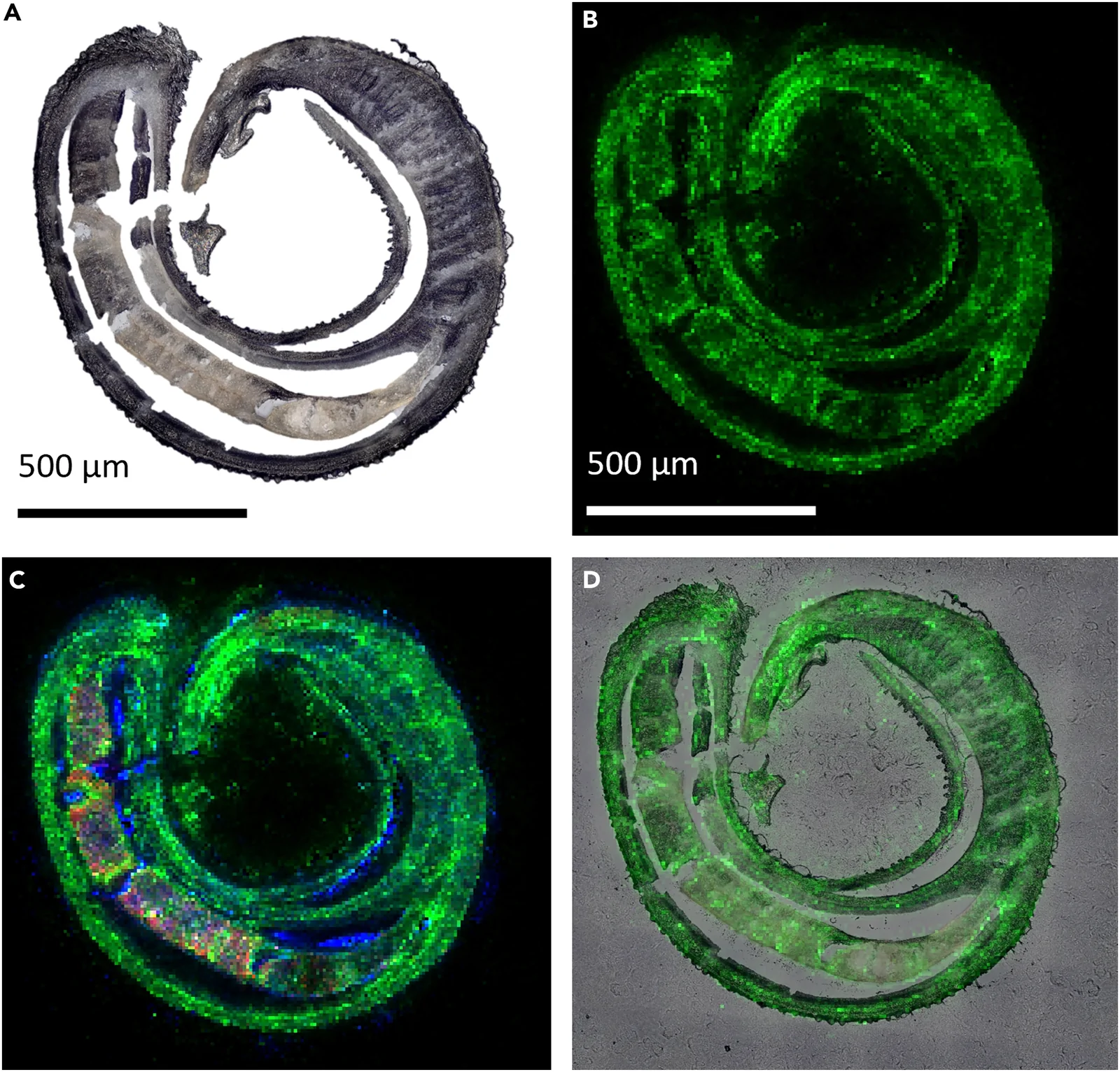
Results of a *Schistosoma mansoni* measurement (A) Optical image of a *Schistosoma mansoni* worm couple, with the female inside of the male’s gynaecophoric canal. (B) MS image of the distribution of the drug Imatinib ([M+H]+, theoretical m/z = 494.2663, measured m/z = 494.2655, deviation 1.70 ppm). (C) Overlayed MS images of the distribution of three different masses (Red: annotated PC O-34:2 [M+H]+, theoretical m/z = 744.5902, measured m/z = 744.5900, deviation −0.21 ppm; Green: Imatinib, [M+H]+, theoretical m/z = 494.2663, measured m/z = 494.2655, deviation −1.70 ppm; Blue: annotated Cholesterol, [M+H-H2O]+, theoretical m/z = 369.3516, measured m/z = 369.3515, deviation −0.31 ppm). (D) Overlayed MS image of Imatinib on the optical image of the worm couple.

**Figure 8:**
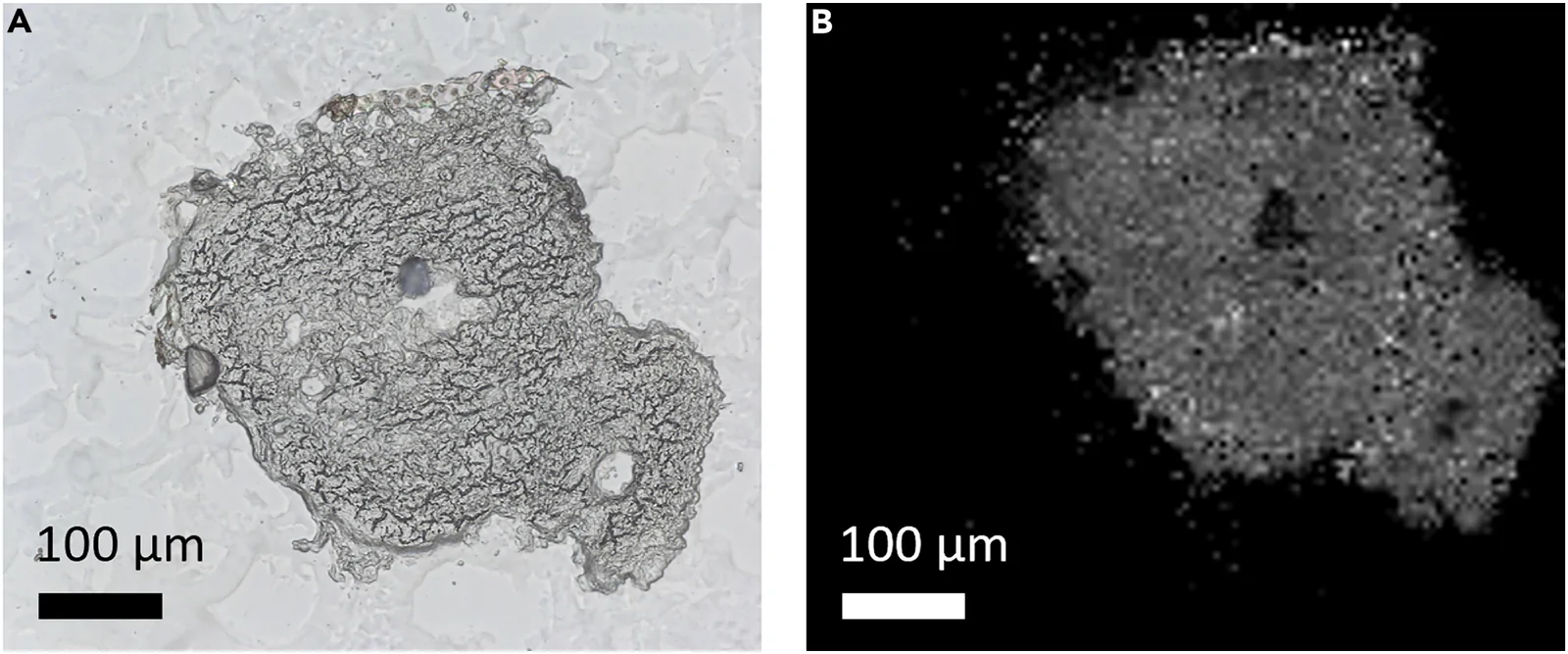
Results of a measurement of *Drosophila melanogaster* brains (A) Optical image of a Drosophila melanogaster brain section. (B) MS image of the distribution of PC 36:4 ([M+H]^+^, theoretical *m*/*z* = 782.5694, measured *m*/*z* = 782.5684, deviation −1.28 ppm, putative annotation based on exact mass).

By applying a gelatin plateau, samples can be completely sectioned with high precision and no sample remains unused. With conventional methods and commercial sample holders, a small amount of the sample must remain on the holder, since the blade cannot cut infinitely close to it. For small samples, this is a problem, since even small amounts of sample inaccessible to analysis can leave out a significant percentage of the sample. Conventional embedding methods such as embedding in gelatin using a cryomold do not work well for small samples, as they rely on the correct orientation of the sample in the gelatin block as well as the correct orientation of the gelatin block itself, Kadesch *et al.* demonstrated this for *S. mansoni* samples.^16^ Using a gelatin plateau ensures the correct orientation of the sample, enabling the production of consecutive whole cross-sections. This is especially important for small samples, as they are often very thin and do not provide enough material to change the orientation of the sample during the sectioning without losing a large portion of the sample. These advantages make the sectioning process more robust, more reproducible, faster and easier. Complete sample loss due to problems during sectioning is highly unlikely (Figures 7 and 8).

A homogeneous application of matrix on such samples requires careful optimization of matrix spraying settings. With the optimized spraying protocol presented, the user should be able to apply it with little or no adjustment to their individual samples.

Since tiny samples tend to produce height differences even within sections due to tissue characteristics changing over relatively short distances, this protocol and the 3D surface measurement capability with the AP-SMALDI^5^ AF ion source enables users to cope with challenging sample topologies.

The software workflow using the Mirion software package enables readers to quickly browse the results of their MALDI MSI measurements, supporting both targeted and untargeted analysis approaches.

## Limitations

While this protocol provides a reliable workflow for the sample preparation and high-resolution measurements of small samples, it has some limitations, especially regarding sample size and mass spectrometry imaging measurements.

Fragile samples that cannot be transferred to the gelatin plateau either via the use of forceps or the micropipette, may not be suitable for use with this protocol, unless another transfer method is used and adapted to this workflow. Another limitation might be the size of the specimen researched; the samples must be small enough to fit on the gelatin plateau. At the same time, the sample must not be too small to be transferred to the gelatin plateau; this limitation might be overcome by adapting the transfer workflow. This protocol is optimized for *S. mansoni* parasites as well as dissected *D. melanogaster* brains and may need adaptation for other sample types, like organoids.

MSI measurements can be influenced by environmental changes, for example, temperature or humidity, affecting the accuracy and reliability of these measurements to some degree. This problem also applies to the matrix deposition, which also needs freshly prepared solutions to minimize contamination. Consistency in preparation of the samples and solutions, conditions in the measurement area, as well as the measurement parameters is important and can minimize these limitations.

## Troubleshooting

### Problem 1

There are air bubbles in the gelatin dome (related to step 25).

### Potential solution

To prevent air bubbles in the gelatin dome you can use a slightly larger amount of gelatin and stop applying the gelatin before the micropipette is empty. To remove air bubbles from the gelatin dome, you can draw them up using a micropipette.

### Problem 2

Gelatin does not freeze in the cryotome (related to step 26).

### Potential solution

The gelatin might have become a supercooled liquid. Lower the temperature in the cryotome further to initiate crystallization or gently touch one side of the gelatin dome with a glass pipette to provide a nucleation site to initialize crystallization.

### Problem 3

Two *S. mansoni* pairs are entangled with each other (related to step 34).

### Potential solution

Use the featherweight forceps to carefully separate the worm couples. This can be aided by using a microscope to obtain a better view of the worm couples. Using a second pair of forceps to stabilize or hold the worms can aid their separation.

### Problem 4

Male and female *S. mansoni* worms are separated, for example due to extended drug incubation (related to step 34).

### Potential solution

Take either a male or a female worm and place it into the water on the prepared glass slide. Afterwards, proceed as described, but you will only measure one sex of the worms.

Alternatively, take a male and a female worm and place them both in the water on the prepared glass slide. Afterwards, place the male on the gelatin plateau after removing the water with a tissue. Directly afterwards, place the female worm next to the male. If necessary, cool the sample carrier down between placing the male and the female. After placing both worms on the gelatin plateau, continue with step 39.

### Problem 5

The *D. melanogaster* brains are not aspirated together with the gelatin (related to step 44).

### Potential solution

The brains might cling to the surface of the glass slide. Gently touch the brains with the pipette tip before transferring them and place the pipette tip directly adjacent to the brain. You may need a microscope to accurately position the pipette relative to the brain. Be careful not to damage the fragile samples.

### Problem 6

Problems encountered during cryosectioning, e.g., cracks in the section, the sample smears during sectioning, the section tears apart completely, or the section gets stuck to the section transfer plate (related to step 52).

### Potential solution

The different problems that can occur during the sectioning process can mostly be solved by simple means.

• Cracks in the section: If you experience cracks in your section, try to increase the section thickness or change the blade, since small dents in the blade can cause cracks in the section. Other approaches include adjusting the speed of the sectioning process as well as the position of the section transfer plate.
• Smearing sample: If you experience sample smearing, lower the temperature in the cryotome to reduce smearing.
• Section tears apart completely: If your section completely tears apart during sectioning, try adjusting the position of the section transfer plate or the speed of the sectioning. If these methods do not work, use tape as an alternative.^22^
• Section gets stuck to the section transfer plate: If your section sticks to the section transfer plate, clean the cryotome including blade and section transfer plate with ethanol.

### Problem 7

The sprayer is clogged (related to step 67).

### Potential solution

Thoroughly clean the sprayer by flushing it with solvent at high flow (e.g., 30 mL/min) for at least 30 min. The solvent composition should be adjusted in terms of polarity to the substance that is causing the clogging. Additionally, clean the spray-head by filling up a 2 mL microcentrifuge tube with 2 mL of solvent and tape it to the bottom of the spray head, submerging the spray tip in the solvent for at least 30 min.

### Problem 8

Matrix crystals are too large; their size should be the same or smaller than the chosen step size during MALDI measurement (related to step 67).

### Potential solution

Changing the spray flow can influence the crystal size, so try to reduce the spray flow if your matrix crystals are too large. If the effect is not sufficient, sublimation of the matrix is another possible solution to achieve smaller crystals.^23^

### Problem 9

The autofocus of the ion source does not recognize the sample surface at some points (related to step 79).

### Potential solution

Three-dimensional surfaces can show uneven matrix crystallization at sharp edges, which can lead to transparent or uncovered sections. Spray a thicker layer of matrix, e.g., 20% more matrix, to ensure complete and homogeneous coverage even at sharp edges of a three-dimensional sample.

## Resource availability

### Lead contact

Further information and requests for resources and reagents should be directed to and will be fulfilled by the lead contact, Prof. Bernhard Spengler (bernhard.spengler@anorg.chemie.uni-giessen.de).

### Technical contact

Technical questions on executing this protocol should be directed to and will be answered by the technical contacts, Annika Sabine Mokosch (annika.s.mokosch@anorg.chemie.uni-giessen.de). and Max A. Müller (max.a.mueller@transmit.de).

### Materials availability

This study did not generate new unique reagents.

### Data and code availability

The datasets shown in this protocol are available from the lead contact upon request.

## Acknowledgments

The authors would like to acknowledge Prof. Christoph G. Grevelding and PD Simone Haeberlein (Justus Liebig University Giessen) for providing *Schistosoma mansoni* samples and Prof. Gero Miesenboeck (University of Oxford) for providing *Drosophila melanogaster* samples. Financial support by the 10.13039/501100001659German Research Foundation (Sp314/23-1, INST 162/500-1 FUGG) and the Hessian 10.13039/100015526Ministry of Science and Education (LOEWE Center DRUID) is gratefully acknowledged.

## Author contributions

A.S.M. optimized the sample preparation protocol for *Schistosoma mansoni*. M.A.M. adapted and optimized the sample preparation protocol for *Drosophila melanogaster* samples. A.S.M. wrote the first draft of the manuscript and prepared all figures. A.S.M., M.A.M., S.G., and B.S. edited the manuscript to its final form. B.S. supervised the project.

## Declaration of interests

M.A.M. and S.G. are employees of and B.S. is a consultant for TransMIT GmbH.
